# Incidence of postoperative dyspepsia is not associated with prophylactic use of drugs

**DOI:** 10.1590/1516-3180.2014.1324676

**Published:** 2014-05-20

**Authors:** Sara Yumi Tsuchie, Fernando Souza Nani, Joaquim Edson Vieira

**Affiliations:** I Resident. Anesthesiology Program, Hospital das Clínicas (HC), Faculdade de Medicina da Universidade de São Paulo (FMUSP), São Paulo, Brazil; II MD. Anesthesiologist, Anesthesia Division, Hospital das Clínicas, Faculdade de Medicina da Universidade de São Paulo (FMUSP), São Paulo, Brazil; III MD, PhD. Associate Professor, Department of Surgery, Faculdade de Medicina da Universidade de São Paulo (FMUSP), São Paulo, Brazil

**Keywords:** Omeprazole, Ranitidine, Postoperative care, Prevention and control [subheading], Dyspepsia, Omeprazol, Ranitidina, Cuidados pós-operatórios, prevenção & controle, Dispepsia

## Abstract

**CONTEXT AND OBJECTIVE:**

Preoperative fasting guidelines do not recommend H2 receptor antagonists or proton pump inhibitors. This study investigated prophylactic use of gastric protection and the incidence of dyspeptic symptoms in the immediate postoperative period.

**DESIGN AND SETTING:**

Non-randomized observational investigation in a post-anesthesia care unit.

**METHODS:**

American Society of Anesthesiologists risk classification ASAP1 and ASAP2 patients over 18 years of age were evaluated to identify dyspeptic symptoms during post-anesthesia care for up to 48 hours, after receiving or not receiving prophylactic gastric protection during anesthesia. History of dyspeptic symptoms and previous use of such medications were exclusion criteria. The odds ratio for incidence of dyspeptic symptoms with use of these medications was obtained.

**RESULTS:**

This investigation studied 188 patients: 71% women; 50.5% ASAP1 patients. Most patients received general anesthesia (68%). Gastric protection was widely used (n = 164; 87.2%), comprising omeprazole (n = 126; 76.8%) or ranitidine (n = 38; 23.2%). Only a few patients did not receive any prophylaxis (n = 24; 12.8%). During the observation, 24 patients (12.8%) reported some dyspeptic symptoms but without any relationship with prophylaxis (relative risk, RR = 0.56; 95% confidence interval, CI: 0.23-1.35; P = 0.17; number needed to treat, NNT = 11). Omeprazole, compared with ranitidine, did not reduce the chance of having symptoms (RR = 0.65; 95% CI: 0.27-1.60; P = 0.26; NNT = 19).

**CONCLUSION:**

This study suggests that prophylactic use of proton pump inhibitors or H2 receptor antagonists was routine for asymptomatic patients and was not associated with postoperative protection against dyspeptic symptoms.

## Abstract (pt)

**CONTEXTO E OBJETIVO:**

Diretrizes para jejum pré-operatório não recomendam antagonistas dos receptores H2 ou inibidores da bomba de prótons. Este estudo investigou o uso profilático de proteção gástrica e a incidência de sintomas dispépticos no período pós-operatório imediato.

**TIPO DE ESTUDO E LOCAL:**

Estudo observacional não aleatorizado em unidade de recuperação pós-anestésica.

**MÉTODOS:**

Pacientes ASAP1 e ASAP2, classificação de risco da *American Society of Anesthesiologists*, com mais de 18 anos de idade, foram avaliados para identificar sintomas dispépticos durante a recuperação pós-anestésica em até 48 horas, tendo ou não recebido proteção gástrica profilática durante a anestesia. História de sintomas dispépticos e uso prévio de tais medicamentos foram critérios de exclusão. A razão de chances para incidência de sintomas dispépticos com uso desses medicamentos foi obtida.

**RESULTADOS:**

Foram estudados 188 pacientes, 71% mulheres, 50,5% dos pacientes ASAP1. A maioria dos pacientes recebeu anestesia geral (68%). Proteção gástrica foi amplamente usada (n = 164; 87,2%), consistindo de omeprazol (n = 126; 76,8%) ou ranitidina (n = 38; 23,2%). Poucos pacientes não receberam qualquer profilaxia (n = 24; 12,8%). Durante a observação, 24 pacientes (12,8%) relataram alguns sintomas dispépticos, porém sem relação com profilaxia (risco relativo, RR = 0,56; intervalo de confiança, IC 95% 0,23-1,35, P = 0,17; número necessário para tratar, NNT = 11). Omeprazol, comparado à ranitidina, não reduziu a chance de ter sintomas (RR = 0,65; IC 95% 0,27-1,60; P = 0,26; NNT = 19).

**CONCLUSÃO:**

Este estudo sugere que o uso profilático de inibidores da bomba de prótons ou antagonistas do receptor H2 foi rotina em pacientes assintomáticos e não esteve associado com proteção pós-operatório para dispepsia.

## INTRODUCTION

There is a strong level of evidence showing that use of histamine (H2) receptor antagonists and proton pump inhibitors reduces gastric volume and acidity in the perioperative period.^1^ In fact, use of proton pump inhibitors has been shown to be an effective treatment, and therefore may be mandatory for preventing upper gastrointestinal diseases after open heart surgery.^2^

Although prophylaxis against stress‐related gastric mucosal lesions is standard in many intensive care units, this practice has been questioned lately, since the reduction in gastric acidity produced by these agents may increase the incidence of infectious gastroenteritis and ventilation-associated pneumonia. It is recommended that drugs altering gastric pH should not be routinely given to critically ill patients without careful consideration.^3^

In relation to practice guidelines for preoperative fasting, a committee of the American Society of Anesthesiologists (ASA) reported that consultants who were selected based on their knowledge of or expertise in preoperative fasting and prevention of pulmonary aspiration disagreed regarding the use of H2 receptor antagonists (91%) or proton pump inhibitors (88%), as routinely administered before elective procedures under general or regional anesthesia, or under sedation, in cases of patients with no apparent increased risk of pulmonary aspiration. The ASA does not recommend such prophylaxis, because of lack of evidence to show that it would reduce the incidence of gastric content aspiration or the associated morbidity and mortality.^1^

The major risk factors for stress ulcers include mechanical ventilation for more than 48 hours and coagulopathy. The minor risk factors include severe sepsis, hypotension or shock, head injury, burns covering more than 25-30% of the body surface area, high doses of steroids, surgery lasting longer than four hours, multiple organ failure, trauma, neurosurgery, quadriplegia, acute renal failure and hepatic failure.^4^ ^-^ ^7^ However, as noted in these studies, stress ulcer prophylaxis has been used even for patients without these risk factors, reaching up to 71% of the patients, which reflects excessive and even inappropriate use of these medications.

Studies addressing stress ulcer prophylaxis have seldom investigated the use of prophylactic medication and its association with dyspeptic symptoms. A search using the terms "Postoperative Care" [Mesh]) and "Premedication" [Mesh] and "Dyspepsia" [Mesh] did not retrieve any results. The setting of Hospital das Clínicas (HC), a tertiary university hospital in São Paulo, Brazil, seemed to be a "natural environment" for this kind of study, considering that anesthesiologists at this hospital routinely use either H2 receptor antagonists or proton pump inhibitors at high rates, for unknown reasons, to promote gastric protection for patients undergoing surgery. 

## OBJECTIVE

The objective of this study was to investigate the prophylactic use of gastric protection and its association with dyspeptic symptoms during the immediate postoperative period.

## METHODS

This study received prior approval from the Research Ethics Committee of Hospital das Clínicas (HC), University of São Paulo School of Medicine (Faculdade de Medicina da Universidade de São Paulo, FMUSP). All patients included in this study gave their informed consent before inclusion. This study was observational and was conducted in a tertiary university hospital with 1,000 operations per month.

Patients at this hospital were evaluated postoperatively in order to identify dyspeptic symptoms. This was done in accordance with the Rome III consensus, defining these symptoms as epigastric pain or heartburn, post-prandial fullness or early satiation. The evaluations were performed while the patients were in the post-anesthesia care unit (PACU), within 48 hours after the operation.^8^ ^,^ ^9^

Data were collected from anesthesia records from August 2010 to March 2011. The patients included were over the age of 18 years and had undergone various surgical procedures with anesthesia. They presented ASA status P1 or P2, and either had or had not received prophylaxis using proton pump inhibitors or H2 receptor antagonists during anesthesia. Patients with a history of dyspeptic symptoms, those who had made previous use of these medications and those who underwent operations under any emergency conditions were excluded. 

The case record files identified the patients' age, sex, ASA status, surgical procedure, type of anesthesia, medication prophylaxis administered, use of nonsteroidal anti-inflammatory drugs (NSAIDs) and presence of dyspeptic symptoms at different times (PACU arrival, 24 hours and 48 hours).

The prevalence of dyspeptic symptoms in patients undergoing a stressful surgical procedure was considered to be unknown, and hence the estimated prevalence was assumed to be 50%. The sample size calculation for 99% confidence interval (z = 2.57) and a sampling error of 10% suggested that the sample size needed to be 165 patients: n = z^2^.[p(1-p)]/error^2^.

The data were presented in descriptive tables, as the mean and standard deviation or as the median and range (minimum-maximum), as appropriate. For age comparisons, analysis of variance (ANOVA) followed by the Sidak test was used. The data were evaluated to ascertain whether they presented normal distribution by means of the Kolmogorov-Smirnov test. The relative risk (RR) with 95% confidence interval (CI) regarding use of chemoprophylaxis and incidence of dyspeptic symptoms was obtained.^10^ Fisher's exact test was used to compare the ASA distribution in relation to gender, and the symptom rate in relation to anesthesia. The tests were performed using the Statistical Package for the Social Sciences (SPSS) for Windows (SPSS Inc., IBM Co., Somers, USA).

## RESULTS

One hundred and eighty-eight patients were evaluated, of whom 134 (71%) were women and 54 (29%) were men; 95 patients (50.5%) were classified as P1 and 93 (49.5%) as P2. There was no difference in distribution of the ASA P classification between men and women (P = 0.75; Fisher). The mean age was 43.3 + 14.8 years, and there was no difference between men and women's ages in this sample (P = 0.093; t test), which showed normal distribution (P = 0.157; Kolmogorov-Smirnov) (Table 1). Most of the patients underwent gynecological (n = 91; 48%), otorhinolaryngological (n = 44; 23%) and urological surgery (n = 37; 20%). The majority underwent balanced general anesthesia (n = 120; 68%) or spinal anesthesia with sedation.

**Table 1:** Characteristics of study population

Gender n (%)	Age range (mean ± SD)	ASA status n (%)	P-value
Male: 54 (29)	18-88 (46.1 ± 18.8)	P1: 69 (51.5%)	
		P2: 65 (48.5%)	
Female: 134 (71)	18-76 (42.1 ± 12.6)	P1: 26 (48.1%)	
		P2: 28 (51.9%)	
Total: 188	18-88 (43.3 ± 14.8)	P1: 95 (50.5%)	0.75
		P2: 93 (49.5%)	

SD = standard deviation

ASA = American Society of Anesthesiologists

The majority of the patients in this sample made prophylactic use of gastric protection (n = 164; 87.2%) with omeprazole (n = 126; 76.8%) or ranitidine (n = 38; 23.2%), and only a few patients did not receive any prophylactic proton pump inhibitor or H2 receptor antagonist (n = 24; 12.8%). During the 48 hours of observation, 24 patients (12.8%) reported some dyspeptic symptoms and, among these, 19 (79.2%) had received prophylactic gastric protectors (Table 2) but did not show any protection in relation to the five patients who had not received any prophylaxis (RR = 0.56; 95% CI: 0.23-1.35; P = 0.17; number needed to treat, NNT = 11). Omeprazole, compared with ranitidine, did not reduce the chance of having symptoms (RR = 0.65; 95% CI: 0.27-1.60; P = 0.26; NNT = 19).

**Table 2:** Dyspeptic symptoms and use of gastric protectors

	Omeprazole	Ranitidine	None	RR (95% CI)
Prophylaxis	126 (67.0%)	38 (20.2%)	24 (12.8%)	
Dyspepsia	13 (10.3%)	6 (15.8%)	5 (20.8%)	0.56 (0.23-1.35)
Anesthesia				
Spinal	38 (30.2%)	18 (47.4%)	12 (50%)	
General	88 (68.8%)	20 (52.6%)	12 (50%)	
NSAID use	84 (44.7%)	29 (15.4%)	13 (6.9%)	
Dyspepsia	8 (9.5%)	6 (20.7%)	3 (23.1%)	0.54 (0.18-1.62)
PACU admission	1 (0.5%)	1 (0.5%)	1 (0.5%)	
24 h	6 (3.2%)	2 (1.1%)	3 (1.6%)	
48 h	6 (3.2%)	3 (1.6%)	1 (0.5%)	

RR = relative risk

CI = confidence interval

**NSAID:** = nonsteroidal anti-inflammatory drug

PACU = post-anesthesia care unit

In the omeprazole group, 38 patients (30.2%) underwent spinal anesthesia while 88 patients (68.8%) received general anesthesia. Ranitidine was given to 18 patients (47.4%) under spinal anesthesia and to 20 patients (52.6%) who received general anesthesia. The patients who did not receive any prophylactic gastric protectors (n = 24) were equally divided between spinal and general anesthesia (n = 12; 50%). The type of anesthesia did not influence the reported complaints during the PACU stay: on arrival (P = 0.55), after 24 h (P = 0.08) or after 48 h (P = 0.53) (Fisher exact test) (Table 2). 

Even among patients who received postoperative analgesia with NSAIDs (n = 126; 67%), use of prophylactic medication did not interfere with the risk of dyspeptic symptoms (RR = 0.54; 95% CI: 0.18-1.62; P = 0.26; NNT = 10) (Table 2).

## DISCUSSION

This study suggested that prophylactic medication for postoperative dyspeptic symptoms was routinely used by anesthesiologists, but that this practice seemed not to provide any protection in comparison with not using prophylaxis.

Most of the data in the literature advocate use of stress ulcer prophylaxis, with greater focus on the risk of gastrointestinal bleeding, especially among intensive care patients. Even among critically ill patients, use of pharmacological prophylaxis is not routinely recommended, because of lack of evidence that this management will result in lower morbidity and mortality. Therefore, this intervention should be advocated only for those at high risk of major bleeding.^4^ ^-^ ^7^

The major risk factors for stress ulcers include mechanical ventilation for more than 48 hours and coagulopathy. The minor risk factors include severe sepsis, hypotension or shock, head injury, burns covering more than 25-30% of the body surface area, high doses of steroids, surgery lasting longer than four hours, multiple organ failure, trauma, neurosurgery, quadriplegia, acute renal failure and hepatic failure.^4^ ^-^ ^7^ However, as noted in these studies, stress ulcer prophylaxis has been used even for patients without these risk factors, reaching up to 71% of our patients, which reflects excessive and even inappropriate use of these medications.

Surgical patients are at greater risk of stress ulcers as a result of hemodynamic disturbances in the visceral circulation, ultimately resulting in mucosal ischemia, changes to the mucous protective barrier, inflammatory conditions and other morbidities.^11^ Studies on neurosurgical patients and children undergoing cardiac surgery for congenital heart disease have shown that prophylactic use of gastric acid suppressants is recommended for high-risk patients, in order to avoid gastrointestinal lesions of greater severity.^12^ ^,^ ^13^ Thus, stress ulcer prophylaxis during the postoperative period is not indicated routinely, but only for those with risk factors for clinically significant bleeding.^11^ ^-^ ^14^

In this study, only a few patients (13%) did not receive prophylactic medication during the study period, thus suggesting that most anesthesiologists at this institution prefer to use these drugs routinely. Although proton pump inhibitor and H2 receptor antagonists have been proven to be safe and well tolerated by most patients, it needs to be borne in mind that there are reports of adverse events in the literature.^10^ ^,^ ^15^ ^-^ ^20^ Although rare, such events could lead to serious morbidity for these patients. In addition, use of these drugs seemed to be widely practiced with poor clinical justification.

There have been reports of allergic reactions ranging from urticaria to anaphylaxis; acute interstitial nephritis; possible drug interaction between clopidogrel and omeprazole, leading to reduction of the effect of the former and subsequently increased cardiovascular risk; interference with bone metabolism and increased risk of bone fracture associated with use of proton pump inhibitors; and increased risk of pneumonia and enteric infections, due to changes in the normal microbial flora of the gastrointestinal tract.^15^ ^-^ ^21^

Although the patients who received drug prophylaxis presented lower risk of dyspeptic symptoms, this result did not reach statistical significance, considering the confidence interval observed. The results also showed seemingly excessive use of these medications. This practice can place unnecessary risk on patients, as well as increasing the financial cost of surgical procedures. On the other hand, gastric protective treatment should not be withheld from patients who genuinely require it.^2^

## CONCLUSION

This study suggests that prophylactic use of proton pump inhibitors or H2 receptor antagonists was a routine among asymptomatic patients but was not associated with protection against dyspeptic symptoms during the postoperative period.
